# Predictors of disengagement from Early Intervention in Psychosis services

**DOI:** 10.1192/bjp.2018.91

**Published:** 2018-08

**Authors:** Francesca Solmi, Abdolali Mohammadi, Jesus A. Perez, Yasir Hameed, Peter B. Jones, James B. Kirkbride

**Affiliations:** 1 Research Associate, Division of Psychiatry, University College London, UK; 2 Specialty Doctor in Psychiatry, St. Ann's Hospital, London, UK; 3 Consultant Psychiatrist, Department of Psychiatry, University of Cambridge, UK, Cambridgeshire and Peterborough Foundation Trust and National Institute for Health Research Collaboration for Leadership in Applied Health Research and Care East of England, UK; 4 Honorary Lecturer, Norfolk and Suffolk Foundation Trust, UK; 5 Professor of Psychiatry, Department of Psychiatry, University of Cambridge, UK, Cambridgeshire and Peterborough Foundation Trust and National Institute for Health Research Collaboration for Leadership in Applied Health Research and Care East of England, UK; 6 Reader in Epidemiology, Division of Psychiatry, University College London, UK.

**Keywords:** Early intervention services, psychosis, cohort study, Social Epidemiology of Psychosis in East Anglia, SEPEA

## Abstract

**Background:**

The effectiveness of Early Intervention in Psychosis (EIP) services for individuals with a first episode of psychosis (FEP) could be thwarted by high rates of early disengagement.

**Aims:**

To investigate which factors predict disengagement with EIP services.

**Method:**

Using data from a naturalistic cohort of 786 EIP clients in East Anglia (UK), we investigated the association between sociodemographic and clinical predictors and disengagement using univariable and multivariable Cox proportional hazards models.

**Results:**

Over half (54.3%) of our sample were discharged before receiving 3 years of EIP care, with 92 (11.7%) participants discharged due to disengagement. Milder negative symptoms, more severe hallucinations, not receiving an FEP diagnosis, polysubstance use and being employed were associated with greater disengagement.

**Conclusions:**

Our findings highlight heterogeneous reasons for disengagement with EIP services. For some patients, early disengagement may hinder efforts to sustain positive long-term EIP outcomes. Efforts to identify true FEP cases and target patients with substance use problems and more severe positive symptoms may increase engagement.

**Declaration of interest:**

None.

Early Intervention in Psychosis (EIP) services offer phase-specific pharmacological, psychological, social, occupational and educational support to individuals with early or prodromal symptoms of psychosis for up to 3 years.^1^ In England, EIP services typically consist of a multidisciplinary team of experts, including psychiatrists, care coordinators, clinical psychologists, psychological therapists and employment and educational specialists.^2^ They were introduced following demonstration that longer duration of untreated psychosis was associated with adverse clinical, functional and social outcomes.^3^ Over the past 15 years, they have gained increased traction worldwide.^4^ In the UK, studies suggest that EIP services can be effective in halting the transition to psychosis^5^ and are cost-effective.^6^^,^^7^ Nevertheless, concerns exist that high rates of early disengagement from EIP services – estimated to range from 13 to 31%^8^^–^^11^ – could hamper their effectiveness, particularly with respect to sustaining long-term positive outcomes. Although studies evaluating the optimal length of EIP service provision have yielded conflicting results,^12^^–^^15^ there is some evidence that a longer time spent in EIP is associated with better long-term outcomes.^14^ It is therefore important to identify potential factors – positive or negative – that predict discharge from EIP services before receipt of a full package of care. Two recent literature reviews found that male gender, unemployment, substance use, not having a family member involved in treatment and belonging to an ethnic minority are the most consistently reported predictors of patient disengagement from EIP services.^16^^,^^17^ However, the evidence is mixed for other factors such as age, symptom severity and social functioning, as they appear to be associated differently with disengagement from pharmacological therapy versus psychosocial services. For example, studies have shown that lower social functioning and symptom severity as well as older age are associated with disengagement from psychosocial services, whereas the reverse is true for pharmacological treatments.^16^^,^^17^ In this study, we sought to investigate sociodemographic and clinical factors that predicted early discharge due to disengagement from six EIP services in the East Anglia region of the UK, using a large, longitudinal data set from the Social Epidemiology of Psychosis in East Anglia (SEPEA) study. Based on findings from the previous literature, we hypothesised that participants with greater substance use, fewer symptoms, and who were male, older and from an ethnic minority background would be more likely to disengage from EIP services.

## Method

### Sample

Individuals referred to six EIP services in East Anglia (UK) for a suspected first episode of psychosis (FEP) between 1 July 2009 and 28 March 2013 were eligible to be included in the SEPEA study if they were: 16–35 years old (or 17–35 years in two EIP services: Cambridgeshire North and South), resident in the catchment area, did not have an intellectual disability or an organic basis to the disorder, and had not been previously in contact with health services for FEP. All participants had suspected psychosis at the time of referral. Details on the characteristics of the catchment area and its representativeness have been provided elsewhere.^18^

All participants were followed up from date of acceptance into EIP services until completion of a 3 year programme of EIP care or discharge from the service, if earlier. In CAMEO North and South the care offering was changed to 2 years for all referrals after 1 October 2013 because of budget constraints. We included all participants accepted onto EIP caseloads, irrespective of later clinical diagnoses (assessed at 6 months following acceptance and at 3 years or discharge, if earlier). In this study, we included all participants who had complete data on the variables of interest. The Cambridgeshire III Local Research Ethics Committee provided ethical approval for this study (09/H0309/39).

### Outcome

Our primary outcome was time to discharge because of disengagement with services. Participants were considered as having disengaged with services after all possible ways to engage them had been explored by the clinical team. These included: appointment letters, phone calls, text messages, emails, home visits and contact with family, friends and other health, education and social care providers. This process involved several attempts (usually at least six to eight attempts over a 2–3 month period). Once all options to re-engage a participant had been exhausted, a decision was made at a clinical team meeting to discharge the person to their general practitioner (GP). The person was then informed of this decision by letter, with a final offer to contact the service if they wished to re-engage. Their GP was also informed of this decision.

Participants who were discharged for other reasons – e.g. because of recovery and transfer to primary care, transfer to a different EIP or mental health service, having moved out of the area or for other reasons (including death, transfer to criminal justice, discharge requested by the participant or drug-induced psychosis) – were censored at their discharge date, but were not considered as having the outcome of interest. All other participants (40.6%) were followed until receipt of 3 years of standard EIP care, including a handful of participants (*n* = 43, 5.4%) who received EIP care for longer than 3 years (median time in care of 39.7 months, interquartile range (IQR) of 37.9–41.4).

### Predictors

We included several sociodemographic and clinical factors previously associated with disengagement from EIP services as potential predictors of disengagement. Sociodemographic data were recorded during the first clinical contact with participants, using a standardised form. We included data on age at referral (16–24, 25–29 and 30–35), gender, ethnicity (White or Black and minority ethnic group, the latter including Black African, Black Caribbean, other Black and minority ethnic groups, Bangladeshi or Pakistani), country of birth (UK or outside the UK), marital status (married or divorced/separated/single) and socioeconomic status (professional, managerial or intermediate; routine or manual; student; unclassified or unemployed).

Participants received an ICD-10 (1992) diagnosis derived by using a two-stage diagnostic procedure comparing clinical and research diagnoses. Clinicians provided a diagnosis at 6 months after EIP acceptance and at discharge. Subsequently, a trained panel of 25 clinicians derived research-based diagnoses from clinical records using 90 standardised symptom items of the Operational Criteria Checklist for Psychotic Illness and Affective Illness (OPCRIT),^19^ a diagnostic instrument known to provide reliable ICD-10 diagnoses (*k* = 0.70).^20^ We defined and grouped OPCRIT-confirmed diagnoses as follows: schizophrenia and other non-affective psychoses (ICD-10 codes F20–F29), substance-induced psychoses (ICD-10 codes F10–F19), bipolar disorder and psychotic depression (ICD10 codes F30–F33) or, if there was no indication of FEP, any other non-psychotic psychiatric diagnosis.

Using auxiliary items from OPCRIT, we included data on presence of a family history of schizophrenia or any other mental health disorders, lack of insight (yes/no), substance abuse and dependency (none, one, two or more substances), experience of an acute psychosocial stressor prior to admission and duration of untreated illness (DUI) (≤1 month, 2–3 months, 4–6 months, 7–12 months or >1 year). DUI was defined as the time elapsed from the emergence of prodromal symptoms (including any active phases of the illness during which non-drug interventions such as cognitive–behavioural therapy may have been received, if those therapies were given because of their potentially antipsychotic properties) to the receipt of the first antipsychotic medication. If the participant did not receive medication, the DUI end-point was defined as time of first non-pharmacological treatment, discharge or time of the 6-month OPCRIT assessment, whichever came sooner. Finally, we included seven standardised indicators of symptom severity (mania, depression, delusions, hallucinations, paranoia, psychomotor poverty and first-rank delusion) following a factor analysis of OPCRIT items to identify psychopathology dimensions in this sample, derived from OPCRIT data. The methodology employed to derive these symptom scores has been described elsewhere (Kirkbride J.B., personal communication 2018).

Finally, we employed two variables to indicate waiting time prior to EIP acceptance, consistent with mandated Access and Waiting Time standards in England (≤2 weeks or >2 weeks^2^) and EIP service (CAMEO North, CAMEO South, West Norfolk EIP, Central Norfolk EIP, Great Yarmouth and Waveney EIP and the former Suffolk EIP).

### Data analyses

Participants contributed to follow-up time from date of EIP acceptance until the end of the 3-year care package (2 years in CAMEO services for patients discharged after 1 October 2013) or discharge, if sooner. We used Chi-square tests and analysis of variance to describe the study sample and length of time in treatment by reason for discharge (‘treatment completed’, ‘left EIP for other reasons’ or ‘disengaged with EIP’).

Next, we investigated sociodemographic and clinical predictors of disengagement using Cox proportional hazard models. Participants who were discharged early from EIP for reasons other than disengagement (*n* = 375, 47.7%) were right-censored and so contributed follow-up time to the analyses where they were potentially ‘at-risk’ of early disengagement. We ran univariable regression models for each potential predictor included in the study, after checking that the proportionality-of-hazards assumption was met. For each univariable model, we estimated Wald-test *P*-values and Akaike's information criteria (AIC) goodness-of-fit parameters. Age, gender, marital status and EIP centre were retained as *a priori* confounders. All other variables were retained for inclusion in multivariable models if they had *P*-values ≤0.05. We employed a forward-fitting modelling approach, progressively including variables with larger AIC values to the multivariable model and testing superiority of each model, compared with the former, via likelihood ratio test (LRT). In *post hoc* analyses, we tested whether the effect of any psychotic symptoms which predicted risk of disengagement differed by the presence or absence of comorbid substance misuse, via LRT for statistical interaction, as before. As a sensitivity analysis, we ran the same models but excluded participants who did not complete the EIP care package for reasons other than disengagement from the analysis. We also re-ran the analyses using a logistic regression model (outcome: 0 = completed treatment package/left for any reason other than disengagement, 1 = disengaged) as a sensitivity analysis, since this did not include length of time in treatment as an assumption. All analyses were performed using Stata version 13.^21^

## Results

### Sample

We included 786 participants in our sample, representing 98.5% of the whole SEPEA study sample (*N* = 798) accepted into EIP care. A total of 12 (1.5%) people were excluded because of missing data on country of origin (*n* = 1), discharge date (*n* = 1), discharge reason (*n* = 2) or marital status (*n* = 8). The overall median time under EIP care was 25.5 months (IQR = 13.3–35.9).

As shown in Table 1, the majority of the sample was male, younger than 25 years, of White British ethnicity, born in the UK, single/divorced/separated, unemployed, had a diagnosis of schizophrenia or other non-affective psychosis, used one or more drugs, had waited longer than 2 weeks to enter treatment and had over 6 months of untreated illness.

**Table 1:** Sample characteristics by disengagement from EIP care

	Type of disengagement from EIP				
	Completed treatment *n* (%)	Left EIP for other reasons *n* (%)	Disengaged with EIP *n* (%)	*P*-value (χ^2^)	Univariable hazard ratio (95% CI)
Disengagement	319 (40.6)	375 (47.7)	92 (11.7)		
Gender					
Female	117 (36.7)	129 (34.4)	25 (27.2)	0.2	Ref
Male	202 (63.3)	246 (65.6)	67 (72.8)		1.45 (0.92–2.30)
Age					
16–19	98 (30.7)	115 (30.7)	27 (29.3)	0.8	Ref
20–24	110 (34.5)	141 (37.6)	31 (33.7)		0.95 (0.57–1.60)
25–29	67 (21.0)	77 (20.5)	18 (19.6)		0.96 (0.53–1.73)
30–35	44 (13.8)	42 (11.2)	16 (17.4)		1.40 (0.75–2.59)
Ethnicity					
White British	253 (79.3)	283 (75.5)	70 (76.1)	0.06	Ref
White other	18 (5.6)	47 (12.5)	7 (7.6)		1.13 (0.52–2.47)
Black	18 (5.6)	10 (2.7)	6 (6.5)		1.53 (0.66–3.51)
Other Black and minority ethnic	19 (5.9)	26 (6.9)	6 (6.5)		1.13 (0.49–2.60)
Bangladeshi/Pakistani	11 (3.4)	9 (2.4)	3 (3.3)		1.26 (0.40–3.99)
Country of Birth					
UK	284 (89.0)	311 (82.9)	82 (89.1)	0.05	Ref
Outside UK	35 (11.0)	64 (17.1)	10 (10.9)		0.96 (0.50–1.88)
Marital status					
Married	34 (10.7)	32 (8.5)	5 (5.4)	0.2	Ref
Single/divorced/separated	285 (89.3)	343 (91.5)	87 (94.6)		1.89 (0.77–4.65)
Employment					
Student/employed	104 (32.6)	170 (45.3)	46 (50.0)	<0.0001	Ref
Unemployed	215 (67.4)	205 (54.7)	46 (50.0)		0.59 (0.39–0.89)*
Social class					
Managerial/intermediate	59 (18.5)	92 (24.5)	17 (18.5)	0.29	Ref
Routine/manual	131 (41.1)	136 (36.3)	41 (44.6)		1.29 (0.74–2.28)
Student	64 (20.1)	85 (22.7)	19 (20.7)		1.08 (0.56–2.07)
Unclassified/unemployed	65 (20.3)	62 (16.5)	15 (16.3)		0.98 (0.49–1.96)
Diagnosis					
No FEP	22 (6.9)	64 (17.1)	22 (23.9)	<0.0001	2.29 (1.41–3.72)*
Non-affective psychosis (F20–29)	244 (76.5)	258 (68.8)	63 (68.5)		Ref
Affective psychoses (F30–33)	45 (14.1)	34 (9.1)	4 (4.4)		0.41 (0.14–1.12)
Substance-induced psychosis (F10–19)	8 (2.5)	19 (5.0)	3 (3.2)		1.06 (0.33–3.38)
Number of drugs used					
None	167 (52.4)	186 (49.6)	31 (33.7)	0.02	Ref
One	70 (21.9)	76 (20.3)	23 (25.0)		1.74 (1.01–2.98)*
Two or more	82 (25.7)	113 (30.1)	38 (41.3)		2.30 (1.43–3.70)*
Family history of schizophrenia					
No	277 (86.8)	321 (85.6)	79 (85.9)	0.89	Ref
Yes	42 (13.2)	54 (14.4)	13 (14.1)		1.05 (0.58–1.88)
Family history mental health					
No	181 (56.7)	224 (59.7)	51 (55.4)	0.63	Ref
Yes	138 (43.3)	151 (40.3)	41 (44.6)		1.09 (0.72–1.64)
Acute psychosocial stressor					
No	216 (67.7)	231 (61.6)	57 (62.0)	0.22	Ref
Yes	103 (32.3)	144 (38.4)	35 (38.0)		1.16 (0.76–1.77)
Insight					
No	227 (71.2)	286 (76.3)	76 (82.6)	0.06	Ref
Yes	92 (28.8)	89 (23.7)	16 (17.4)		0.61 (0.35–1.04)
Waiting time					
Up to 2 weeks	153 (48.0)	186 (49.6)	40 (43.5)	0.57	Ref
More than 2 weeks	166 (52.0)	189 (50.4)	52 (56.5)		1.16 (0.77–1.76)
EIP centre					
CAMEO North	44 (13.8)	53 (14.1)	7 (7.6)	<0.0001	Ref
CAMEO South	70 (21.9)	86 (22.9)	18 (19.6)		1.46 (0.61–3.50)
West Norfolk	10 (3.1)	30 (8.0)	6 (6.5)		1.76 (0.59–5.23)
Central Norfolk	80 (25.1)	44 (11.7)	22 (23.9)		1.78 (0.76–4.18)
Great Yarmouth and Waveney	52 (16.3)	31 (8.3)	9 (9.8)		1.15 (0.43–3.08)
Suffolk	63 (19.8)	131 (34.9)	30 (32.6)		1.95 (0.86–4.44)
Duration untreated illness					
Up to 4 weeks	24 (7.5)	59 (15.7)	6 (6.5)	0.01	Ref
5–8 weeks	16 (5.1)	24 (6.4)	12 (13.0)		3.28 (1.23–8.74)*
9–12 weeks	17 (5.3)	21 (5.6)	3 (3.3)		0.89 (0.22–3.57)
3–6 months	55 (17.2)	50 (13.3)	13 (14.1)		1.29 (0.49–3.38)
6–12 months	89 (27.9)	94 (25.1)	23 (25.0)		1.33 (0.54–3.26)
Over 12 months	118 (37.0)	127 (33.9)	35 (38.1)		1.51 (0.63–3.59)

	Mean (s.d.)	Mean (s.d.)	Mean (s.d.)	*P*-value	Univariable hazard ratio (95% CI)
Manic symptoms	0.09 (0.9)	−0.05 (1.1)	−0.08 (0.8)	0.11	0.86 (0.69–1.06)^a^
Depressive symptoms	0.04 (1.0)	−0.03 (0.9)	−0.05 (1.0)	0.62	0.93 (0.76–1.14)^a^
Delusions	0.10 (0.9)	−0.09 (0.9)	−0.006 (1.1)	0.03	0.96 (0.78–1.17)^a^
Hallucinations	−0.02 (1.0)	−0.06 (1.1)	0.32 (0.9)	0.004	1.35 (1.10–1.67)^a^*
Paranoia	0.03 (1.1)	−0.03 (0.9)	0.03 (0.9)	0.05	1.00 (0.82–1.22)^a^
Psychomotor poverty	0.05 (1.1)	0.01 (0.9)	−0.22 (0.9)	0.06	0.79 (0.65–0.95)^a^*
First-rank delusions	0.09 (1.0)	−0.01 (1.0)	−0.37 (0.8)	0.0005	0.71 (0.59–0.86)^a^*

EIP, Early Intervention in Psychosis; Ref, reference; FEP, first episode of psychosis.

a Change in risk (hazard) of being discharged due to disengagement associated with 1 s.d. change in symptoms.

* *P* ≤ 0.05.

### Rate and predictors of disengagement

A total of 467 (59.4%) participants were discharged early from EIP services in our region (Table 2). Of these participants, 40 (5.1%) were transferred to EIP services outside the region (meaning that at least 54.3% of our sample were discharged before receiving a full EIP care package) and 92 (11.7%) were discharged due to disengagement, our primary outcome. Other reasons for discharge ae shown in Table 2 and in a flow chart presented in Supplementary Figure 1 available at https://doi.org/10.1192/bjp.2018.91. Participants who were discharged because they had moved out of the area had the shortest follow-up time (median 5.6 months, IQR 3.8–10.4). Participants who left the programme due to disengagement had a median follow-up time of 15.0 months (IQR 8.2–21.2) (Table 2).

**Table 2:** Proportion of participants who completed and did not complete (with reason for discharge) full EIP care as well as median stay (months) in treatment with interquartile range (*N* = 786)

	*n* (%)	Median duration of treatment
Completed 3 years of EIP care	319 (40.6%)	35.9 (35.1–36.0)
Disengagement (primary outcome)	92 (11.7%)	15.0 (8.2–21.2)
Moved out of area	34 (4.3%)	5.6 (3.8–10.4)
No FEP	27 (3.4%)	12.3 (8.5–26.3)
Recovery	150 (19.1%)	15.8 (10.3–25.6)
Transferred to GP	14 (1.8%)	20.0 (14.7–20.7)
Transferred to another EIP	40 (5.1%)	8.9 (4.0–18.1)
Transfer to another mental health service	56 (7.1%)	16.5 (8.9–23.4)
Other^a^	54 (6.9%)	22.7 (14.1–28.3)

EIP, Early Intervention in Psychosis; FEP, first episode of psychosis; GP, general practitioner.

a Including participants who were stable, who requested to be discharged, who had substance-induced symptoms, who died or who were under the care of the criminal justice system

Univariable analyses (Table 1) showed that participants who were not diagnosed with FEP were more likely to be discharged due to disengagement compared with those diagnosed with non-affective psychosis. Participants in employment, with one or polysubstance misuse/dependency and those who had greater hallucinatory symptoms were also at greater risk of being discharged early from EIP services due to disengagement. Univariable analyses also suggested that participants with more severe manic symptoms, severe psychomotor poverty and first-rank delusions and a diagnosis of affective psychosis were at reduced risk of disengagement. Disengagement did not vary by other characteristics, including ethnicity, EIP service and family history of mental illness (Table 1).

Following multivariable modelling (Table 3), we found evidence that participants who did not receive an FEP diagnosis (hazard ratio 2.52, 95% CI 1.49–4.26), who had a DUI of between 5 and 8 weeks (hazard ratio 5.19, 95% CI 1.85–14.56) and who had a history of polysubstance misuse (≥2 drugs; hazard ratio 2.20, 95% CI 1.30–3.72) were at greater risk of disengagement from EIP services. Those who were unemployed (hazard ratio 0.44, 95% CI 0.28–0.69), with more severe psychomotor poverty (hazard ratio 0.71, 95% CI 0.57–0.88) and first-rank delusions (hazard ratio 0.74, 95% CI 0.60–0.83) were at lower risk of disengagement. We also found weak evidence that men (hazard ratio 1.61, 95% CI 0.98–2.63, *P* = 0.06), participants with more hallucinations (hazard ratio 1.24, 95% CI 0.97–1.58, *P* = 0.08), those aged 30–35 years (hazard ratio 1.88, 95% CI 0.95–3.70, *P* = 0.07) and those with single substance misuse (hazard ratio 1.66, 95% CI 0.94–2.94, *P* = 0.08) were at increased risk of disengagement. In *post hoc* analyses, we considered whether the increased risk of disengagement associated with more hallucinations differed between those with and without comorbid substance misuse: there was weak evidence that more severe hallucinations were associated with risk of disengagement among people without comorbid substance misuse (hazard ratio 1.64, 95% CI 1.12–2.40) but not in those with a history of substance misuse (hazard ratio 1.06, 95% CI 0.79–1.43, LRT *P* = 0.069).

**Table 3:** Multivariable Cox proportional hazard model

	Multivariable hazard ratio (95% CI)^a^
Gender	
Female	Ref
Male	1.61 (0.98–2.63)*
Marital Status	
Married	Ref
Single, divorced or separated	1.55 (0.58–4.13)
Age	
16–19	Ref
20–24	1.17 (0.68–2.01)
25–29	1.28 (0.67–2.45)
30–35	1.88 (0.95–3.70)*
EIP centre	
CAMEO North	Ref
CAMEO South	1.94 (0.79–4.78)
West Norfolk	1.70 (0.55–5.20)
Central Norfolk	1.05 (0.42–2.64)
Great Yarmouth and Waveney	0.88 (0.30–2.53)
Suffolk	1.31 (0.55–3.13)
Duration of untreated illness	
Up to 4 weeks	Ref
5–8 weeks	5.19 (1.85–14.56)**
9–12 weeks	0.78 (0.19–3.210)
3–6 months	1.56 (0.58–4.19)
6–12 months	1.51 (0.59–3.86)
Over 12 months	1.64 (0.67–4.02)
Employment	
Student/employed	Ref
Unemployed	0.44 (0.28–0.69)**
Diagnosis	
No FEP	2.52 (1.49–4.26)**
Non-affective psychosis (F20–29)	Ref
Affective psychoses (F30–33)	0.39 (0.14–1.13)*
Substance-induced psychosis (F10–19)	0.66 (0.19–2.22)
Number of drugs used	
None	Ref
One	1.66 (0.94–2.94)*
Two or more	2.20 (1.30–3.72)**
Psychomotor poverty^b^	0.71 (0.57–0.88)**
First-rank delusions^b^	0.74 (0.60–0.83)**
Hallucinations	1.24 (0.97–1.58)*

Ref, reference; EIP, Early Intervention in Psychosis; FEP, first episode of psychosis.

a Hazard ratio above one indicates greater likelihood of being discharged.

b Change in risk (hazard) of being discharged due to disengagement associated with 1 s.d. change in symptoms.

*0.1 > *P* > 0.05, **P ≤ 0.05.

In sensitivity analyses we re-ran our models, excluding anyone discharged early for reasons other than disengagement. This provided similar results (data available from authors), although the risk of disengagement became stronger with male gender (hazard ratio 1.70, 95% CI 1.03–2.80) and hallucinations (hazard ratio 1.28, 95% CI 1.00–1.64). Results were similar when using logistic regression models, ignoring time in treatment (data available from authors).

## Discussion

### Main findings

This is the first epidemiological study in the UK to investigate sociodemographic and clinical predictors of disengagement from EIP services using a large naturalistic cohort sample. We found that over half of all clients accepted into EIP services in the East of England (54.3%) did not complete 3 years of care, which may have considerable implications for achieving sustained long-term positive outcomes following early intervention. Nonetheless, the total number of clients discharged due to disengagement was lower (11.7%). Disengagement was associated with being employed (or a student), not meeting diagnostic criteria for FEP during EIP care, having a history of polysubstance misuse or dependency, having lower levels of psychomotor poverty and fewer first-rank delusions.

### Meaning of the findings and comparisons with previous studies

Previous studies have found a proportion of service disengagement ranging between 13^8^ and 31%.^9^ Although our study broadly aligns with these figures, differences in the definition of disengagement used across studies make direct comparisons difficult.^22^^,^^23^ For instance, we did not include patients who moved out of the catchment area (*n* = 34, 4.33%) in our measure of disengagement; whereas one previous study did,^10^ reporting a disengagement level of 24% within 12 months of admission. If we used the same definition, albeit over a 3-year period, we would have seen a total of 16% of patients disengaging. This suggests that disengagement from EIP services in the East of England may have been lower than previously found.

Nonetheless, Doyle *et al*^22^ and Lai and Malla^23^ have suggested that the heterogeneity in reported disengagement rates may partly result from the absence of an internationally recognised standard to define disengagement from EIP care. We note that other studies have used broad definitions of disengagement: discontinuation of EIP care despite on-going need^8^ (13% patients disengaged), failure to present to services despite contact being made by case manager^11^ (23% patients disengaged), not making contact with EIP for three consecutive months (28% disengagement)^24^ and leaving the programme prior to the end of the 30 months of treatment (31% patients disengaged).^9^ The latter definition appears to be the broadest and it aligns closely with the rate of disengagement in our study when defined in this way (34%), having excluded those who were discharged due to recovery. It is also possible that heterogeneity in these estimates reflects differences in healthcare systems in different countries; with the exception of a single trial,^25^ no study has been conducted in the UK. The lack of consistency in definition of disengagement from EIP services may hinder effective clinical service provision and makes comparability across research studies difficult. We suggest that policy guidance should be developed to allow clinicians and services to more accurately monitor and minimise service disengagement.

Despite differences in disengagement rates, our findings in relation to predictors of severity are largely in agreement with the previous literature, including lower symptom severity associated with greater disengagement.^8^^–^^11^^,^^26^ However, in contrast to earlier studies (which mainly used scales of overall^8^^,^^11^^,^^26^ or positive and negative^9^^,^^10^ symptoms), we found strong evidence of disengagement in participants with lower psychomotor poverty (i.e. negative symptoms) and fewer first-rank delusions (i.e. a positive symptom). Although this encouraging finding suggests that participants with more severe psychopathologies are successfully retained in EIP, it nonetheless highlights that a proportion of people with milder initial presentations, or those who do not later meet diagnostic criteria for FEP, may be accepted onto EIP caseloads. This raises the possibility that those who present with less severe psychopathologies may be more likely to disengage early, as evidenced in our study among those in employment at baseline. These results are also consistent with the possibility that early intervention care resulted in symptom remission in some individuals, facilitating later disengagement. Although these participants may also have been false-positive presentations to EIP services, who may require signposting to other specialist mental health services, it is also possible that they represent a group of high-risk individuals for whom EIP care has been successful during the period of observation.

Research has consistently found greater disengagement rates among individuals with a history of substance misuse^10^ or who continue to use drugs while in treatment.^11^ This mirrors our observations: we found that patients with substance misuse or dependency (including alcohol) issues were more likely to disengage with services, with some evidence of a dose-response association between number of substances used and risk of disengagement. We also found that participants reporting more severe hallucinations were more likely to disengage, with this association being stronger in sensitivity analyses. Here, *post hoc* analyses provided some weak evidence that this effect was more pronounced in people without substance use than in those with comorbid substance misuse. Although this finding warrants further attention, it tentatively suggests that individuals with comorbid mental health or behavioural issues have additional needs which may result in less disengagement with EIP services. This may be because EIP services have more intensive or regular contact with participants that have more complex morbidities. Unfortunately, we did not have information on the extent (i.e. frequency and quality) of engagement prior to discharge/disengagement, but our findings suggest this is an important area for future research. Nonetheless, over half of our sample had no documented history of substance misuse and those with more hallucinations had a higher risk of disengagement, underlining the need for EIP services to meet the heterogeneous needs of clients to prevent premature disengagement.

Finally, the high overall discharge rate (54.3%) (*vis-à-vis* disengagement) highlights that only a minority of people accepted on caseloads complete the full EIP care package. Although several studies have shown that positive effects of EIP are often not sustained after patient discharge,^13^^,^^15^^,^^27^ a recent trial has found some evidence that individuals assigned to extended EIP – lasting 1 year longer than the usual care package – may have better long-run outcomes in terms of social functioning (e.g. full-time employment rate).^15^ This suggests that longer engagement with EIP care could result in greater long-term sustainability of its positive effects and to the need for greater effort to ensure patient retention in EIP care programmes. However, we also acknowledge that length of engagement is only one of several indices that are likely to determine clinical and social outcomes following EIP care: quality of engagement is also highly relevant and it is important that engagement also results in a meaningful therapeutic alliance. As Lai and Malla^23^ have suggested, meaningful engagement is only likely to occur routinely when services can align their care offering to not only be efficacious but also ‘caring, respectful, and nonjudgmental’. Further, they suggest clinicians ‘need to be flexible in how, when, and where services are delivered, and which components of services are delivered over time.’ We echo their call that disengagement should also be regarded as a multidimensional construct and that people may disengage from services in a variety of ways, or may only engage with certain aspects of care. All such issues will affect the quality of engagement and instruments that can assess such issues need to be developed, validated and deployed in future studies.^28^^,^^29^

### Methodological considerations

This study had a number of strengths. We employed data from a large naturalistic cohort of EIP clients across six sites, representing 98.5% of all those people who met the inclusion criteria for the SEPEA study. At initial presentation and acceptance to services we included people who were later found not to meet a diagnosis of psychotic disorder, allowing us to identify this as a reason for disengagement. This has potential implications for service provision, as it suggests that better identification of ‘at-risk’ cases might result in more targeted and, potentially, cost-effective interventions. We included data on a large number of predictors, including clinical variables derived from OPCRIT, and raters were trained, showing good interrater reliability.^18^ However, several clinical predictors were derived from single OPCRIT items, which may have resulted in some degree of measurement error. Furthermore, although extensive, we acknowledge that the list of predictors we could investigate did not include some factors that have previously been associated with disengagement from EIP services. For instance, although not unequivocally,^24^ evidence suggests that individuals who do not have a family member involved with treatment^9^ or who are not living with family^11^^,^^26^ disengage with EIP more frequently. In our study, we found weak evidence of greater disengagement among unmarried participants in univariable models, but we did not have data on family involvement. A recent review also highlighted that different pathways to care in individuals who experience FEP could be associated with greater stigma, poorer help-seeking attitudes and adherence to treatment.^30^ In our study we did not have information on pathways to care, although one previous study did not find evidence that this affected disengagement.^24^ We also did not have information on levels of social functioning and had limited power to explore associations with disengagement for some of our predictors (e.g., marital status and DUI), which could be proxies of functioning and is reflected in the imprecise estimates reported here. Our definition of early discharge due to disengagement was based on clinical information, which might have contained a degree of error. Nonetheless, for all participants discharged early, exhaustive methods were implemented to retain clients where possible and we carefully checked all cases for inconsistencies in coding, finding only one instance of misclassification.

## Supplementary material

For supplementary material accompanying this paper visit https://doi.org/10.1192/bjp.2018.91. click here to view supplementary material
