# Supplementary figures and images for: Predictors of disengagement from Early Intervention in Psychosis services
Source: Br J Psychiatry. 2018 Aug;213(2):477–83. doi: 10.1192/bjp.2018.91 (PMC6071847; doi:10.1192/bjp.2018.91)

**Supplemental Material**

**Figure S1: Flowchart of study sample**

**
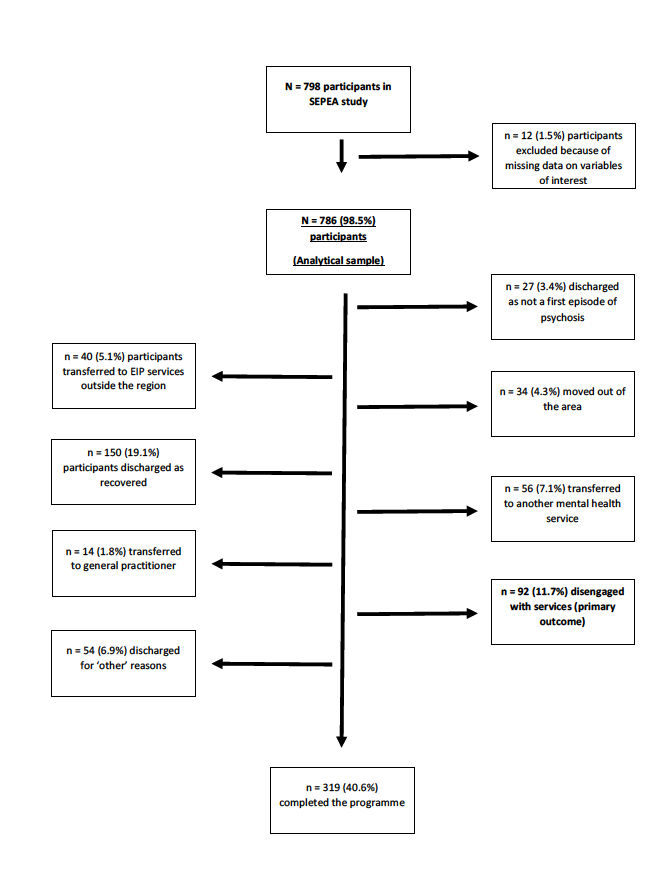
**

Supplement: Supplementary file 1 [file S0007125018000910sup001.docx]
